# Supplementary material for: Development of Dl1.72, a Novel Anti-DLL1 Antibody with Anti-Tumor Efficacy against Estrogen Receptor-Positive Breast Cancer
Source: Cancers (Basel). 2021 Aug 13;13(16):4074. doi: 10.3390/cancers13164074 (PMC8392387; doi:10.3390/cancers13164074)
Supplement: Supplementary file 1 [file cancers-13-04074-s001.zip › cancers-1301791-supplementary.pdf]

Supplementary Materials:

Development of Dl1.72, A Novel Anti-DLL1 Antibody with Anti-Tumor Efficacy Against Estrogen Receptor-Positive Breast Cancer

Gabriela Silva, Joana Sales-Dias, Diogo Casal, Sara Alves, Giacomo Domenici, Clara Barreto, Carolina Matos, Ana R. Lemos, Ana T. Matias, Khrystyna Kucheryava, Andreia Ferreira, Maria Raquel Moita, Sofia Braga, Catarina Brito, M. Guadalupe Cabral, Cristina Casalou, Duarte C. Barral, Pedro M. F. Sousa, Paula A. Videira, Tiago M. Bandeiras and Ana Barbas

Methods

Surface Plasmon Resonance

D11.72 was immobilized on a Protein A (Series S) sensor chip via the Fc region at 25 ºC. HBS-P+, consisting of 10 mM HEPES, pH 7.4, 150 mM NaCl, 0.05% Tween-20, was used as the background buffer during immobilization. D11.72 was diluted to 0.5 µg/mL in HBS-P+ and coupled to the surface for 1 and 2 min injection time at a flow rate of 10 µL/min, generating 100 and 200 response units (RU), respectively. rhDLL1 was directly diluted in running buffer (HBS-P+) and tested at 6 different concentrations using a 2-fold dilution series, with the highest concentration tested being 32 nM. Interaction analysis cycles consisted of a 220-sec sample injection (30 µL/min; association phase) followed by 600-sec of buffer flow (dissociation phase). All sensorgrams were processed by first subtracting the binding response recorded from the control surface (reference spot), followed by subtraction of the buffer blank injection from the reaction spot. All datasets were fit to a simple 1:1 Langmuir interaction model to determine the kinetic rate constants. Experiments were performed on a Biacore 4000 (Cytiva, Uppsala, Sweden) at 25 ºC and evaluated by the provided Biacore 4000 Evaluation software.

Horseradish Peroxidase Anti-DLL1 Dl1.72 Conjugation

Two mg of Dl1.72 and Ctr Ab were conjugated with a 4-fold molar excess of horseradish peroxidase (HRP) using the EZ-Link™ Plus Activated Peroxidase kit (Thermo Scientific, #31488) as per manufacturer´s instructions for conjugating activated peroxidase to an antibody at pH 9.4 using BupHTM Carbonate-Bicarbonate Buffer. mAb-HRP conjugates were purified using Pierce Conjugate Purification Kit (Thermo Scientific, #44920). mAb-HRP conjugation was verified by SDS-PAGE and Western blotting in non-reducing conditions as recommended by the supplier´s instructions. The ability of Dl1.72-HRP to specifically bind DLL1 proteins was confirmed by ELISA using 96-wells not coated or pre-coated with rhDLL1-Fc, or Fc proteins.

SDS-PAGE and Western blot analysis

Proteins were resolved by electrophoresis transferred to polyvinylidene fluoride membranes and blotted with rabbit anti-DLL1 polyclonal antibody (Abcam, #ab84620), Protein A peroxidase (Sigma-Aldrich, P8651, for detection of mAbs and Fc proteins), or goat anti-human IgG (Fab specific)-peroxidase antibody (Sigma-Aldrich, A0293) as described in [18]. Blots were developed with the enhanced chemiluminescence substrate western lightning plus ECL reagent (PerkinElmer, NEL103E001EA) and digital images acquired in a Bio-Rad imaging system.

Immunohistochemical Analysis

For the Immunohistochemical (IHC) analysis of human ER+ BC, tissue sections slides were incubated in a humidified chamber for 10 min at 90 ºC and deparaffinized in Xylene for 10 min at room temperature (RT). Rehydration was performed through an ethanol series of 100%, 96%

and 70% (v/v), 2 min each, finishing with ddH2O. Endogenous peroxidase was blocked with 3% (v/v) H2O2 for 10 min. Antigen retrieval was achieved using 10 mM Sodium Citrate with 0.05% (v/v) Tween-20 (pH 6), for 2 min at 120 ºC. Then, several wash steps were performed, namely with ddH2O for 2 min and 2 times with PBST/0.05% for 5 min. To avoid non-specific antibody binding, blocking with 0.5% BSA for 1 h at RT was performed, followed by 3 washes with PBST/0.05% for 5 min each. Incubation with Dl1.72-HRP, Ctr Ab-HRP (7.5 µg/mL each), or anti-DLL1 Ab (Abcam, ab84620, 10 µg/mL) was performed for 1 h at RT in a humidified chamber. After being washed, samples incubated with anti-DLL1 ab84620 were further incubated with peroxidase-conjugated mouse anti-rabbit IgG (Jackson Immunoresearch, #211-032-171) for 1 h at RT in the humidified chamber. Finally, the slides were washed, incubated with 3, 3′diaminobenzidine-hydrochloride (DAB, IL ImmunoLogic, BS04-110) following the manufacturer′s instructions and counterstained with hematoxylin. Slides were mounted using Entellan medium and images were acquired using Axio Imager Z2 microscope (Zeiss, 10x objective).

Immunostaining of mice tumors, livers, and kidney tissue sections was performed by BenchMark ULTRA - Automated IHC and in situ hybridization slide staining systems (Ventana, Roche). Antigen retrieval was performed with an ULTRA CC1 at 94 ºC for 20 min for pan-cytokeratin staining or for 56 min for Ki67. For pan-keratin staining an additional 4 min incubation with 3% (v/v) H2O2 Ultraview universal DAB inhibitor reagent was performed to block endogenous peroxidase. Then, samples were incubated at 37 ºC for 24 min with the pan-cytokeratin AE1/AE3/PCK26 antibody cocktail (760-2135) for the identification of human epithelial cells or anti-human Ki67 (05298512001) for detection of proliferating cells. Detection of the antigen-Ab complex was achieved with the UltraView Universal DAB Chromogen and the UltraView Universal DAB H2O2 substrate for 8 min followed by intensification with the UltraView Universal DAB Copper solution for 4 min. The nuclear contrast was performed with Hematoxylin and Bluing for 4 min each. All used reagents and Abs were from Ventana. Slides were mounted using Bio Mount HM (Bio-optica, Milan Italy, 05-BMHM500) and scanned using NanoZoomer - SQ Digital Slide Scanner (Hamamatsu, Japan).

Supplementary Figures and Tables

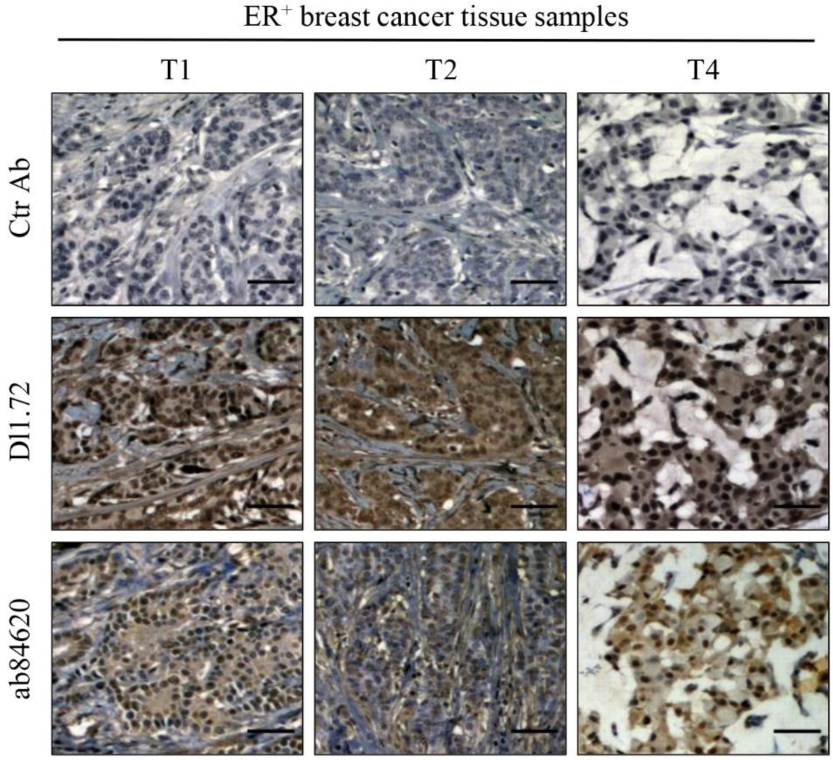

Figure S1. Binding of Dl1.72 to human ER+ BC tumor samples by IHC. Matched-tissue sections from ER+ BC samples (n = 9) were stained individually with Dl1.72 or Ctr Ab, pre-conjugated with HRP, or with the commercial anti-DLL1 polyclonal ab84620 followed by anti-rabbit HRP-conjugated secondary antibody as a control for DLL1 detection and tissue distribution. Representative IHC images from matched samples at disease stages T1, T2, and T4 are shown.

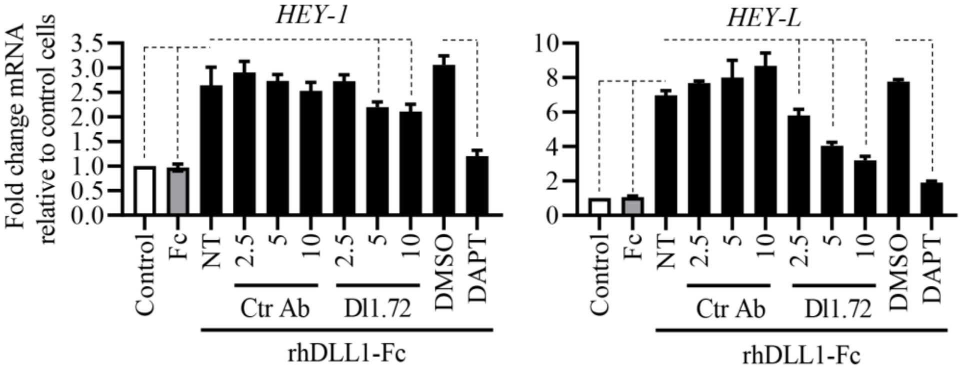

Figure S2. Dose-response effect of anti-DLL1 Dl1.72 in the expression levels of Notch-dependent genes induced by the ligand in MCF-7 cells. Cells were cultured in control non-coated wells or wells pre-coated with Fc or rhDLL1-Fc proteins in the absence (NT) or presence of the indicated concentration (μg/mL) of Dl1.72, negative control isotype antibody (Ctr Ab), the pan-Notch inhibitor DAPT (5 μM), or DMSO (DAPT vehicle). After 17 h, total RNA was isolated, and the transcript levels of *HEY-1* and *HEY-L* were determined by RT-qPCR. Graphs show mRNA fold change relative to control cells (mean ± SD) from triplicates in one out of three independent experiments, with similar results.

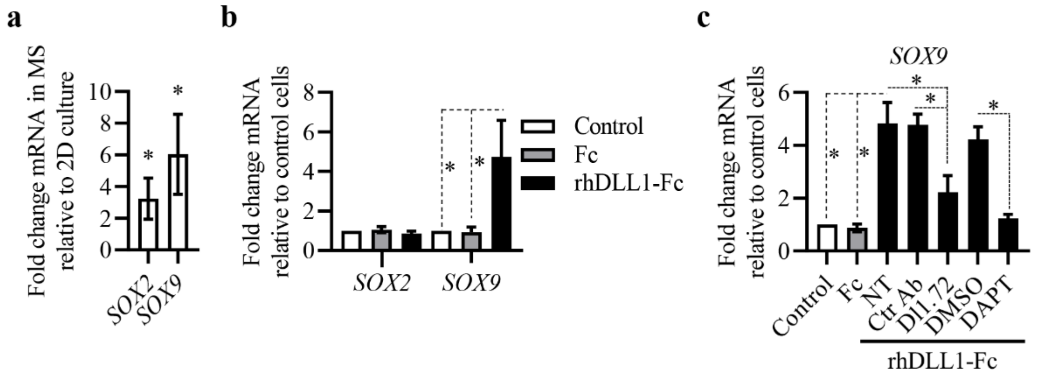

Figure S3. DLL1 induces the expression of the stemness-associated gene *SOX9* in MCF-7 cells and anti-DLL1 Dl1.72 impairs DLL1-mediated *SOX9* expression. **(a)** Validation of increased levels of stemness-associated genes *SOX2* and *SOX9* in control untreated mammospheres in assays from Figure 3 F relative to parental cells grown in parallel in 2D cultures determined by RT-qPCR. **(b)** Effect of DLL1 in the expression levels of *SOX2* and *SOX9* genes in MCF-7 cells. Cells were cultured in non-coated wells (control) or wells pre-coated with rhDLL1-Fc or Fc proteins. After 17-20 h, the transcript levels of *SOX2* and *SOX9* were determined by RT-qPCR. **(c)** Effect of Dl1.72 on DLL1-driven *SOX9* induction. Cells were cultured in control not coated wells or wells pre-coated with Fc or rhDLL1-Fc in the absence (NT) or presence of Dl1.72, Ctr Ab (10 µg/mL each), DAPT (5 µM) or DMSO and *SOX9* expression was determined as above. The graphs show mean ± SD of $n = 3$ (each in triplicate). *, $P < 0.05$ (two-tailed paired Student t-test).

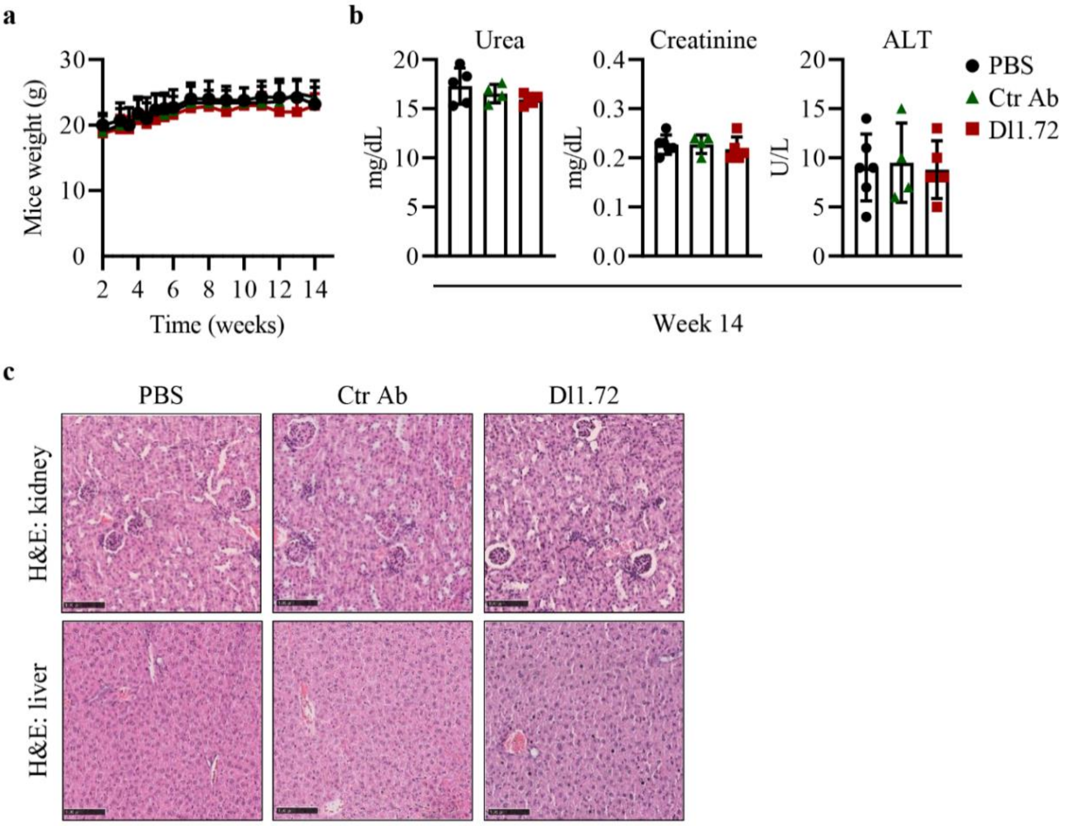

Figure S4. Anti-DLL1 Dl1.72 toxicity and safety assessment in vivo. **(a)** Body weight curves of mice treated with Ctr Ab, Dl1.72 ($n = 5$ mice/each group) or PBS ($n = 6$ mice) for 12 weeks. **(b)** Values of urea, creatinine, and ALT (alanine aminotransferase) in the sera of mice treated as indicated after 12 weeks of treatment. All graphs show mean ± SEM. **(c)** Representative images of cross-sectioned kidneys and livers stained with H&E of mice from each experimental group after 12 weeks of treatment, showing no tissue damage. Scale bars, 100 µm.

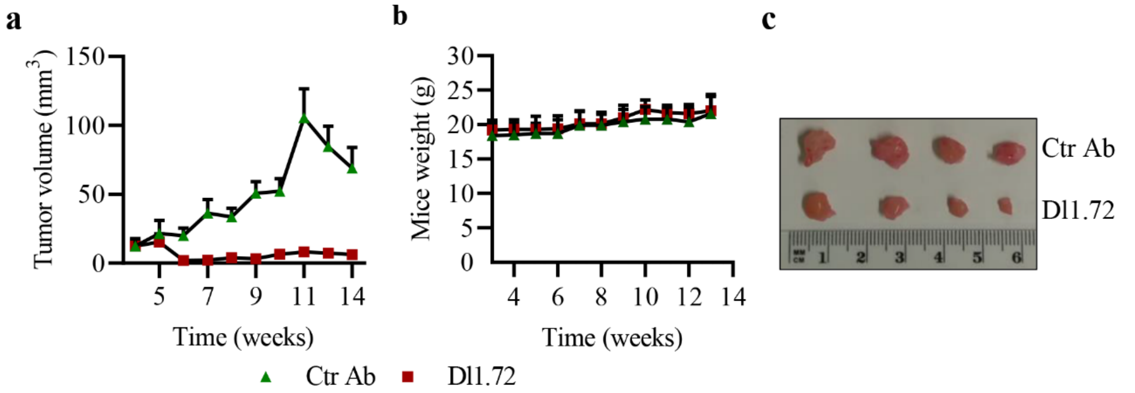

Figure S5. Second experiment to validate tumor growth inhibition by anti-DLL1 Dl1.72 in a MCF7 xenograft mice model. Four days after subcutaneous injection with17β-estradiol, NSG mice were inoculated with 2 x 10^6 MCF-7 cells in 100 µl of PBS:matrigel (1:1 ratio, 2 mammary fat pad injections per mice). After fifteen days, mice were injected intraperitoneally with either Dl1.72 or isotype-matched Ctr Ab at 10 mg/Kg twice a week for 12 weeks (n = 10 mice/group). Tumor volume **(a)** and mice body weight **(b)** were determined weekly. **(c)** Representative whole tumor images from each group after 12 weeks of treatment immediately after resection. * P < 0.05 (Two-way ANOVA with Sidak multiple comparison test).

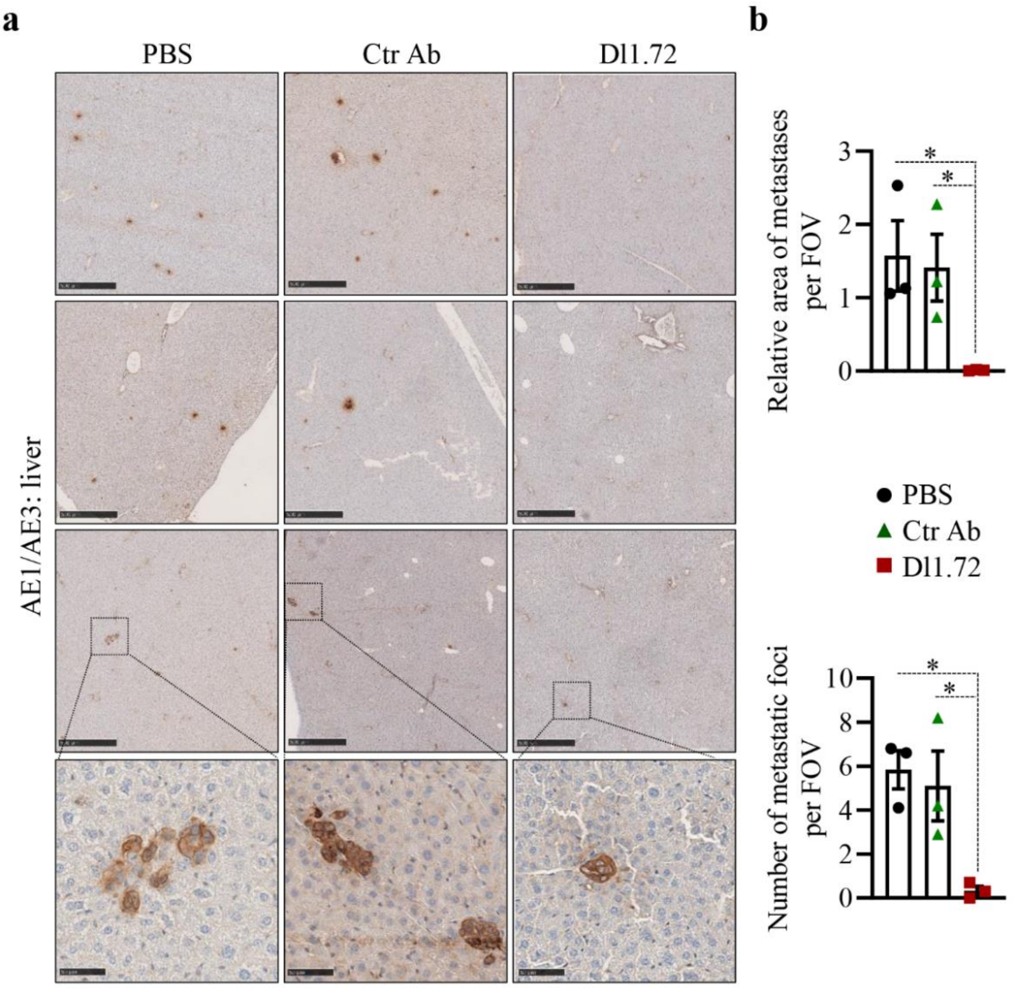

Figure S6. Anti-DLL1 Dl1.72 impairs the occurrence of BC liver micrometastases in a MCF-7 xenograft mice model. **(a)** Representative IHC images of cross-sectioned livers from mice treated with either PBS, Ctr Ab or Dl1.72 twice weekly for 12 weeks stained with pan-cytokeratin AE1/AE3 Ab specific to human epithelial cells. Scale bars, 2.5 mm (top image) and 100 µm (bottom image). **(b)** The graphs show quantification of liver relative areas (mm^2) occupied by AE1/AE3$^+$ cells and number of liver micrometastatic foci (i.e. number of

AE1/AE3+ areas) of indicated groups (mean ± SEM, *n* = 3/group) per field of view (FOV). Quantifications were done in ten random fields for each mouse liver. *, P < 0.05 (Mann–Whitney test).

Table S1. Recombinant Notch ligands and control proteins used for plate coating in ELISA.

Protein	Source (catalogue number/reference (Ref.))
Recombinant human DLL1-Fc	Generated in house (rhDLL1-ECD-Fc in Ref. 1)
Recombinant human JAG1-Fc	Generated in house (rhJAG1-ECD-Fc in Ref. 1)
Recombinant human DLL3	R&D Systems (9749-DL-050)
Recombinant human DLL4 Fc	R&D Systems (10185-D4-050)
Recombinant human JAG2 Fc	R&D Systems (1726-JG-050)
Recombinant mouse DLL1 Fc	R&D Systems (5026-DL-050)
Fc control protein	Generated in house (Ref. 1)
His-tag control protein	Generated in house

Ref. 1- *N Biotechnol* (2021) doi:10.1016/j.nbt.2021.05.003

Table S2. Primers used for transcript amplification.

Target		Sequence (5´- 3´)
HEY-1	forward	GTTCGGCTCTAGGTTCCATGT
	reverse	CGTCGGCGCTTCTCAATTATT
HEY-L	forward	GGAAGAAACGCAGAGGGATCA
	reverse	CAAGCGTCGCAATTCAGAAA
HPRT1	forward	CCTGGCGTCGTGATTAGTGAT
	reverse	AGACGTTCAGTCCTGTCCATAA
RPL22	forward	CACGAAGGAGGAGTGACTGG
	reverse	TGTGGCACACCACTGACATT
SOX2	forward	GCGGAAAACCAAGACGCT
	reverse	ATGTGCGCGTAACTGTCCAT
SOX9	forward	AGACCTTTGGGCTGCCTTAT
	reverse	TAGCCTCCCTCACTCCAAGA

Table S3. Clinicopathological characteristics of breast cancer (BC) tissue samples obtained from the pathology department of Hospital Professor Doutor Fernando Fonseca (HFF).

Sample Number	Sex	Age at Diagnosis (Years)	BC	Tumor	Node	Histological Grade
1	Female	37	ER^+	T4	Nx	III
2	Female	85	ER^+	T1	N1	II
3	Male	63	ER^+	T1	N1	II
4	Female	66	ER^+	T1	N0	I
5	Female	71	ER^+	T1c	N1	II
6	Female	79	ER^+	T2	N2	III
7	Female	76	ER^+	T2	N1	II
8	Female	82	ER^+	T3	N0	II
9	Female	65	ER^+	T4	N+	III

ER^+ - estrogen receptor-positive; T - tumor; N - node; x - nodes unable to be assessed
